# Amino-Terminal Fusion of Epidermal Growth Factor 4,5,6 Domains of Human Thrombomodulin on Streptokinase Confers Anti-Reocclusion Characteristics along with Plasmin-Mediated Clot Specificity

**DOI:** 10.1371/journal.pone.0150315

**Published:** 2016-03-14

**Authors:** Neeraj Maheshwari, Satish Kantipudi, Anand Maheshwari, Kashika Arora, Vandana, Neha Kwatra, Girish Sahni

**Affiliations:** CSIR-Institute of Microbial Technology, Chandigarh, India; King Abdullah International Medical Research Center, SAUDI ARABIA

## Abstract

Streptokinase (SK) is a potent clot dissolver but lacks fibrin clot specificity as it activates human plasminogen (HPG) into human plasmin (HPN) throughout the system leading to increased risk of bleeding. Another major drawback associated with all thrombolytics, including tissue plasminogen activator, is the generation of transient thrombin and release of clot-bound thrombin that promotes reformation of clots. In order to obtain anti-thrombotic as well as clot-specificity properties in SK, cDNAs encoding the EGF 4,5,6 domains of human thrombomodulin were fused with that of streptokinase, either at its N- or C-termini, and expressed these in *Pichia pastoris* followed by purification and structural-functional characterization, including plasminogen activation, thrombin inhibition, and Protein C activation characteristics. Interestingly, the N-terminal EGF fusion construct (EGF-SK) showed plasmin-mediated plasminogen activation, whereas the C-terminal (SK-EGF) fusion construct exhibited ‘spontaneous’ plasminogen activation which is quite similar to SK i.e. direct activation of systemic HPG in absence of free HPN. Since HPN is normally absent in free circulation due to rapid serpin-based inactivation (such as alpha-2-antiplasmin and alpha-2-Macroglobin), but selectively present in clots, a plasmin-dependent mode of HPG activation is expected to lead to a desirable fibrin clot-specific response by the thrombolytic. Both the N- and C-terminal fusion constructs showed strong thrombin inhibition and Protein C activation properties as well, and significantly prevented re-occlusion in a specially designed assay. The EGF-SK construct exhibited fibrin clot dissolution properties with much-lowered levels of fibrinogenolysis, suggesting unmistakable promise in clot dissolver therapy with reduced hemorrhage and re-occlusion risks.

## Introduction

Blood clot or thrombus formation within the vascular system is a life-saving process only when it occurs subsequent to a natural hemorrhage. But thrombus formation, or its subsequent migration, can be life-threatening when it occurs either inside the blood vessels such as that of the brain (causing thrombotic strokes), or the coronary artery- causing heart attacks.

Most preferred and available thrombolytics such as streptokinase (SK), Urokinase (UK) and tissue-type Plasminogen Activator (tPA) or its engineered derivatives [1–3], have been widely used against various circulatory disorders. All essentially operate through a broadly similar mechanism wherein they catalyse the scission of the scissile peptide bond between residues Arg561-Val562 of plasminogen, and convert it into its proteolytically activated form, plasmin. In last two-three decades, several attempts have been made to construct better versions of streptokinase such as by site-specific mutational manipulation, N- and C-terminal truncation, PEGylation, and fusion derivative construction. A detailed description of these constructs has been summarized in a recent review article [4].

Streptokinase’s mode of action as a protein co-factor has been delineated to occur in two distinct pathways [5]. Pathway I defines the streptokinase and plasminogen (HPG) high-affinity binding and formation of an active complex that is catalytically active but one in which the scissile peptide bond of the ‘partner’ HPG is not yet cleaved but capable to convert free human plasminogen into plasmin (HPN) [6,7]. In pathway II streptokinase directly forms a 1:1 complex with plasmin and this activator complex further acts on ‘substrate’ human plasminogen molecules to produce plasmin exponentially [5,8,9]. Literature indicates that in pathway-I, the N-terminal of SK plays a crucial role in HPG activation. The truncation of 59 residues from the N-terminal resulted in a reduced direct plasminogen activation but increased fibrinolysis and better level of surviving fibrinogen during clot lysis[10]. In another attempt at increasing the specificity of SK action, a Fab fragment of monoclonal antibody specific to fibrin was fused with SK that resulted in a slow but efficient clot lysis[11]. Such modifications that result in selective fibrin lysis but with reduced fibrinogen destruction (i.e. greater fibrin selectivity) are highly desirable clinically, as these results in reduced tendency for internal hemorrhages. If pathway-I of plasminogen activation is suppressed by, say, mutations, such a molecule will not be able to activate HPG even after binding with HPG, but if Pathway-II capability (i.e., formation of an active HPG activator complex with plasmin) survives in the SK mutant, one can expect it to behave as a clot specific HPG activator. This is particularly so since the fibrin clots are plasmin-rich [12] as they are shielded from inhibition by blood alpha-2-antiplasmin, which is rapidly inactivated in the blood stream[13]. Hence, during streptokinase therapy, the side effects due to the high amounts of non-specifically (systemically) generated plasmin, such as hemorrhage, can be avoided using such a SK variant.

It is known that tissue plasminogen activator (tPA) and streptokinase-mediated clot dissolution *in vivo* causes increased thrombin activity that is manifested in elevated levels of fibrinopeptide-A during, and subsequent to, thrombolysis [14,15]. Increased fibrinopeptide-A levels correlate with thrombin generation, and in myocardial infarction as well as ischemic strokes [16], re-occlusion/hypercoagulability in patients receiving thrombolytic therapy continues to be a major concern [17], and even though, during thrombolytic therapy, heparin administration is often prescribed it does not substantively reduce thrombin generation [18]. This is essentially because, during clot lysis, clot-bound thrombin gets released that is resistant towards heparin inhibition. However, the clot-bound thrombin is susceptible towards thrombomodulin [19–21]. Thrombin is also known to be responsible for the activation of a wide range of substrates in the coagulation pathway which amplify the coagulation cascade reaction, like conversion of fibrinogen into fibrin [22], activation of platelets (protease activated receptors are cleaved by thrombin) [23], activation of factor V [24], factor VIII [25], factor XI [26], factor XIII [27], and the activation of thrombin-activable fibrinolysis inhibitor (TAFI) [28].Thus, heparin inhibits free thrombin but does not affect the well-known ‘indirect’ thrombin promoters, namely factor V and factor VIII [29]. Thrombomodulin, or its isolated EGF domains 4, 5, 6, can directly cause thrombin inhibition and also activate Protein C, by redirecting thrombin’s activity by making a complex, that degrades activated forms of factor V and factor VIII [30,31].

In the present study, well-known side effects such as lack of clot specificity of SK, and consequent post-thrombolytic complications such as non-specific proteolysis of fibrinogen leading to increased hemorrhage risk, as well as thrombin-mediated re-occlusion problems have been addressed. This was carried out by designing and preparing new fusion constructs, wherein EGF 4,5,6 domains derived from human thrombomodulin were fused either at the N-terminal or C-terminal end of SK. These fusions were expressed as secretory proteins in *Pichia pastoris* (yeast), and isolated from the exudate, purified to near homogeneity, and characterized with respect to their functional properties. The results (detailed below) show the successful preparation of new, and potentially useful, protein engineered entities that may be of significant clinical benefit.

## Materials and Methods

### Reagents

For DNA cloning purpose, *E*. *coli* XL 1Blue strain was purchased from Stratagene Inc. (La Jolla, CA). Thermostable DNA polymerase (*Pfu*) and the QuikChange^TM^ Site-Directed Mutagenesis Kit were also obtained from Stratagene Inc. Restriction endonucleases, T4 DNA ligase and other DNA modifying enzymes were acquired from New England Biolabs (Beverly, MA). Oligonucleotide primers were supplied by Biobasic, Inc., Canada. Purifications of DNA and extraction of PCR amplified products from agarose gels were performed using kits available from Qiagen GmbH (Germany). Automated DNA sequencing using fluorescent dyes was done on Applied Biosystems 3130xl genetic analyzer with 16 capillaries. Glu-plasminogen was either purchased from Roche Diagnostics GmbH (Penzberg, Germany) or purified from human plasma by affinity chromatography (Deutsch and Mertz, 1970). Urokinase, EACA, sodium cyanoborohydride, and L-Lysine were purchased from Sigma Chemical Co., St. Louis, USA. Phenyl Agarose 6XL (Prometic Inc., Canada) and DEAE-Sepharose™ (Fast Flow) were procured from GE-Health Care Life Sciences, Uppsala, Sweden. Human Protein C, human thrombin, and hirudin were purchased from Merck Millipore Inc., Germany. Recombinant human thrombomodulin (HEK 293 cell line-derived) was purchased from American Diagnostica, MA, USA. The polynucleotide sequence corresponding to the human EGF 4,5,6 domains of thrombomodulin was custom synthesized by GenScript, NJ, USA. All other reagents were of the highest analytical grade available.

### Genetic construction of fusion constructs

#### N-terminal EGF-SK and C-terminal SK-EGF fusion construct preparation

Double-stranded (ds) DNA blocks, encoding for EGF-SK and SK-EGF protein fusion, in which the Epidermal growth factor (EGF) like domains 4,5,6 (oxidation resistant) of thrombomodulin[32,33] were custom-synthesized (Gene Script Inc.).The design and construction of the bacterial expression vector pET 23-d_SK has been described by Nihalani *et al*. (1998) [34]. EGF 4,5,6 encoding sequences were fused in-frame at the N-terminus, and C-terminus of the SK ORF, with the help of Overlap Extension PCR [35,36]. These fusion constructs were then in-frame fused after a nucleotide stretch encoding the *kex-2* enzyme cleavable sequence in the pPIC 9K vector and expressed in SMD 1168 strain of *Pichia pastoris* by using BMGY and BMMY media[33].

### Purification of SK/EGF-SK/SK-EGF fusion constructs

For purification of expressed plasminogen activator proteins, freshly harvested culture supernatant was passed through 0.45μ and 0.22μ filters successively, after which immediately EDTA to final concentration of 10 mM was added (all processing was carried out at 4°C). The culture supernatant was then concentrated about 2–3 fold with 30 KDa TFF cut-off membrane cassettes (tangential flow filtration system; Merck Millipore Inc.) and finally brought to 20 mM sodium acetate containing 10mM EDTA, pH 5.5, buffer in a TFF system. The supernatant was loaded onto a 20-ml Q-Sepharose column which was pre-equilibrated with 20 mM sodium acetate and 10mM EDTA, pH 5.5 (equilibration buffer). After completion of loading, the column was washed ~5 bed-volumes of equilibration buffer for removal of impurities. Elution of bound protein was done by applying an increasing gradient of 0.5 M NaCl prepared in the equilibration buffer over 20 bed-volumes of column matrix. Eluted fractions from Q-Sepharose chromatography, which showed plasminogen activation, were pooled and made 1M in NaCl, 10mM EDTA, and 50 mM phosphate buffer, pH 7.4, and loaded onto pre-equilibrated Phenyl-agarose (20 ml packed beads) column. After loading, column was washed with 5bed-volumes of the high salt equilibration buffer to wash loosely bound impurities. Elution was done by applying a salt-to-water gradient over 20 bed-volumes of column matrix. In the final step, active protein was concentrated by using (30 KDa-cutoff) centrifugal concentrators, and desalted by using Superdex G-25 column.

### Plasminogen activation assays

All chimeric fusion polypeptide constructs contained a thrombolytic component, so capability of these constructs to activate human plasminogen was measured by measuring release of color from chromogenic peptide substrate using a well established one-stage procedure [37].

### Plasmin dependent plasminogen activation assays of SK-EGF fusion constructs

A one-stage assay method [37] was used for the plasminogen activation assay with different EGF and SK fusion constructs (EGF-SK and SK-EGF) as before, but in order to check the plasmin dependency in the various activators, a slightly modified assay was used. In this modified assay, increasing amounts of human plasmin were added into 100 μl reaction mixes that contained 2 μM plasminogen, 50 mMTris-Cl, pH 7.4, 0.05% BSA and 0.5 mM chromogenic substrate, and fixed concentration of one of the constructs/nSK (0.5 nM) which were added the last and release of pNA (p-nitroanilide) was measured at 405 nm continuously for a period of 1 h.

### Determination of plasminogen activation steady-state kinetic parameters of SK, EGF-SK and SK-EGF chimeras

Plasminogen activation kinetic parameters of SK, EGF-SK and SK-EGF chimeras were determined as described [9,38].

### Clotting time experiments

The effect on thrombin-induced clot formation by the presence of different chimeric constructs was done using a modified clotting assay [39].

### Protein C activation assays and determination of kinetic constants

Fusion constructs, EGF-SK and SK-EGF were subjected to thrombin-mediated Protein C activation assays using chromogenic substrates. The protein C kinetic parameters were calculated as described by White *et al*., 1995 [33].

### Comparative fibrin (in presence of clot-bound thrombin) clot lysis by EGF-SK and SK_EGF

In this method, clots were prepared by adding plasminogen-free fibrinogen (final concentration 4 mg/ml) in HBS (hepes buffered saline) 20 mM CaCl_2_, pH 7.4and 1 IU of thrombin was added, and reactions kept at 37°C for 2 h. After that, clots were washed with HBS buffer three times in order to remove excess unbound and non-coagulated fibrinogen. In order to check whether chimeric EGF 4,5,6 domains, fused with SK either at N- or C–termini, are capable of binding with thrombin that was incorporated into fibrin clots and thereby can lyse faster, the washed clots were incubated with SK, EGF-SK or SK-EGF fusion constructs in separate wells at 5 nM final concentration for 1 h. After incubation, clots were washed 3 times with HBS buffer to remove any excess and unbound fusion constructs. Finally, 1μM human plasminogen was added to initiate clot lysis and percentage change in absorbance at 405 nm was monitored over time.

### Fibrinogen estimation in plasma after clot lysis by SK, EGF-SK and SK-EGF fusion constructs

Different concentrations (0.1–1000 nM)of SK, SK-EGF or EGF-SK were added in plasma and incubated at 37°C, and samples were taken out at 15, 30 and 45 min, and their fibrinogen concentrations were determined by the sodium sulfite precipitation procedure, as described [40].

### Clot lysis in plasma by SK, EGF-SK and SK-EGF fusion constructs

In this method, fluorescein-labeled-fibrinogen was used for the detection of clot lysis[41]. In brief, purified fibrinogen was labeled with FITC (fluorescein isothiocyanate dye) (2 mg /ml in DMSO) under alkaline conditions (100 mM sodium carbonate buffer, pH 9.2), where 50 μl dye (2 mg/ml) was added into 10 mg/ml fibrinogen solution, and kept for 1 hr at room temperature followed by 16 h at 4°C. Unreacted dye was removed by desalting (with G-25 Sephadex column, from GE-Amersham) and labeled fibrinogen fractions containing average 11–13 dye molecules/mole of fibrinogen were obtained in the breakthrough volume. In order to prepare fluorescein-labeled clots, 100 μl of fluorescein-labeled fibrinogen (~0.5 mg labeled fibrinogen) was mixed with 450 μl of human plasma after which 5 mM CaCl_2_ (2.5 μl of 1MCaCl_2_) and 4 IU of human thrombin (4 μl) were added, and mix incubated at 37°C for 1 hr. Clots were then washed with HEPES-saline buffer (HBS) 3 times in order to remove unreacted fluorescein-labelled fibrinogen. The fluorescein-clot was incubated in 20 ml of human plasma for 1 h in 50 ml amber-colored falcon tube so as to enrich it with plasminogen and other coagulation factors that mimic *in vivo* like conditions. SK, EGF-SK or SK-EGF fusion constructs were then added, whereby fluorescein-clot lysis was initiated. As clot lysis progressed, labeled fibrin (in clot) gets degraded and released into plasma, and this can be measured as increase in fluorescence intensity in plasma. Maximum fluorescence that can be attained in clot lysis (100% clot lysis) was determined by adding 450 μl of plasma, 6.5 μl water and 100 μl of labeled fibrinogen reaction mixture in 20 ml of plasma and measuring fluorescence, which was considered as 100%.

### Demonstration *in vitro* of re-occlusion, and its prevention by fusion constructs

An *in vitro* re-occlusion model was developed on the basis of available knowledge [42], and tested with the fusion constructs to check the prevention, if any, of clot-bound thrombin-mediated re-occlusion occurring in the controls (into which no fusion construct was added). In this model, three distinct phases were discerned, namely: a) clot formation phase; b) clot lysis phase and c) re-occlusion phase. In brief, 100 μl clots were prepared in flat-bottom microtiter plates by adding plasminogen-free, purified human fibrinogen (final concentration 4 mg/ ml), 20 mM CaCl_2,_ HBS buffer (0.01 M HEPES, 0.13 M NaCl, pH 7.4) and 3 IUof thrombin (3μl), followed by incubation at 37°C for 2 h, during which absorbance changes at 405 nm were periodically recorded to measure clot formation. The clots were then washed 3 times with 100 ul of HBS buffer to remove excess unbound thrombin. Following this, 1 μM human plasminogen, HBS and either SK, EGF-SK or SK-EGF fusion constructs were added (25 nM each), and final volume made up to 100 μl on the fibrinogen clot mesh (100 μl fibrin clot + 100 μl buffer containing plasminogen, buffer and thrombolytic agent). Clot lysis was monitored by recording change (seen as a decrease) in absorbance at 405 nm. At times corresponding to ~ 40% clot lysis, clots were washed 3 times with 100 μl of HBS buffer in order to remove free plasmin. After that, for the initiation of re-occlusion phase, 100 μl of HBS buffer that contained fibrinogen (final concentration 4 mg/ml) and 0.1μM human α-2 anti-plasmin (to inhibit any traces of plasmin, and to mimic an *in vivo* situation) were added, and change (seen as an increase) in absorbance at 405 nm was monitored with time. The reformation of clots after the initial lysis by the thrombolytic/s was considered as the re-occlusion phase. All assays were carried out in triplicates. In this assay, after clot formation, in control experiments, HBS with plasminogen was added without any thrombolytic, for a time period for clot lysis taken by other fusion constructs. Clots were then washed three times with 100 μl of HBS buffer. One set of clots was incubated with 100 μl of HBS and α-2 antiplasmin (0.1 μM), and the other set incubated with 100 μl of fibrinogen (4 mg/ml) containing α-2 antiplasmin (0.1μM) in HBS. The change in absorbance at 405 nm was then measured.

## Results

Fusions of the EGF 4,5,6 domains (oxidation resistant) [33] of human thrombomodulin with streptokinase at either the latter’s N-terminal or C-terminal end using appropriate DNA gene blocks were designed [36]. As mentioned in literature EGF 4,5,6 domains contain 9 disulfide bonds and their proper oxidative refolding is necessary for full functional activity that is not possible in reduced cytoplasmic environment of bacteria whereas mammalian eukaryotic expression systems often provide low yield and are expensive for production; therefore, *Pichia pastoris* seemed the best system for these constructs. Extracellular expression of heterologous protein provides considerable ease of purification/downstream processing because it separates desired protein from a plethora of cytoplasmic proteins. The N-terminal and C-terminal gene constructs were then successfully obtained from *Pichia pastoris* where the encoded polypeptides were secreted into the extracellular milieu [33], and could be purified to a very high level by a simple two-step process.

**Fig 1:**
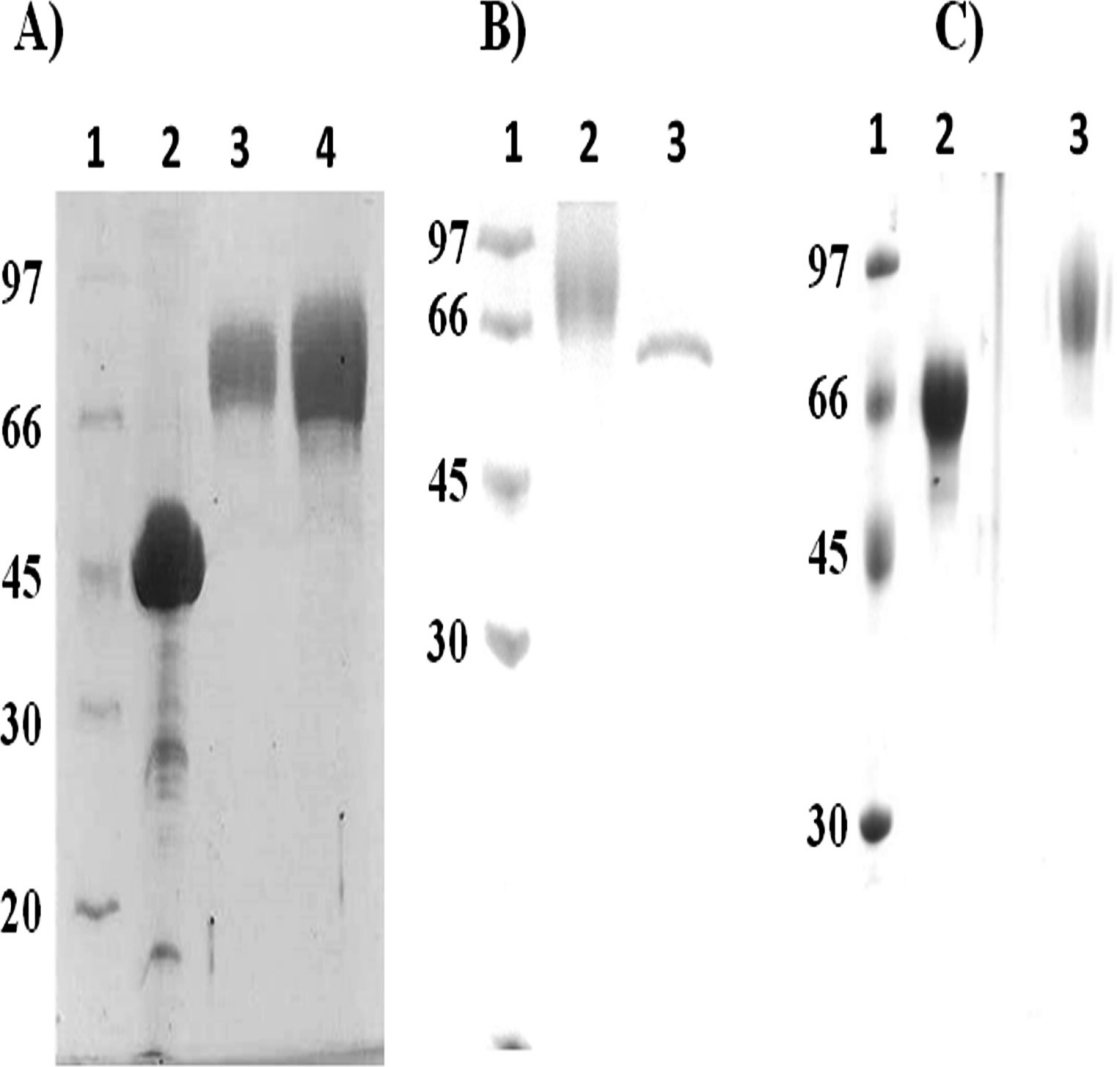
SDS-PAGE analysis of different purified proteins in 10% acrylamide gels. In Fig 1A) Lane 1 shows standard molecular weight markers; lane 2, purified SK with molecular weight of ~ 47 KDa; lane 3, Purified EGF-SK; lane 4, SK-EGF which shows molecular weight above the expected 66 KDa, likely due to *P*. *pastoris* glycosylation in the EGF4,5,6 domains which contains two N-linked glycosylation sites, and the SK moiety of the fusions, containing 3 N-linked glycosylation site (see text). In Fig 1B) lane 1 shows standard molecular weight markers; lane 2, purified SK-EGF and lane 3 showed deglycosylated SK-EGF. In Fig 1C) lane 1shows molecular weight markers; lane 2, deglycosylated EGF-SK, lane 3 shows EGF-SK.

After purification, both the chimeric polypeptides were found to be essentially homogenous (Fig 1) by SDS-PAGE gels, and their molecular weights were around 66 KDa, which is in contrast to their expected theoretical polypeptide masses, namely 59.85 and 56.984 KDa, respectively. A probable reason for their higher molecular weight is the likely presence of glycosylation on both the expressed protein sequences, particularly since *Pichia pastoris* is known to cause mannose-rich glycosylation in expressed proteins [43]. After treating with deglycosylation enzyme (PNGase F; that removes N-linked sugar moieties), their molecular weight uniformly shifted below, to approximately 59KDa (Fig 1B and 1C). *Pichia* mediated glycosylation did not affect the activity (see later, below) of any component of fusion protein hence further experiments were carried out with glycosylated proteins. The amino acid analyzes data of the two constructs were matched with the theoretical composition and found to be in consonance with expectation (see Tables A and B in S1 File). The purified proteins were also subjected to N-terminal protein sequencing. The N-terminal fused EGF 4,5,6-SK (EGF-SK) construct showed a N-terminal sequence as follows: VDPC* FRAN, that corresponds with the 4^th^ domain of human EGF sequence (36), where it was amplified prior to fusion by PCR. The C-terminal SK-EGF construct showed the sequence IAGPEWL at the N-terminus that matches with the known N-terminal sequence of SK derived from *S*. *equisimilis*[44].

### Plasminogen activation characterization of EGF-SK and SK-EGF

**Fig 2:**
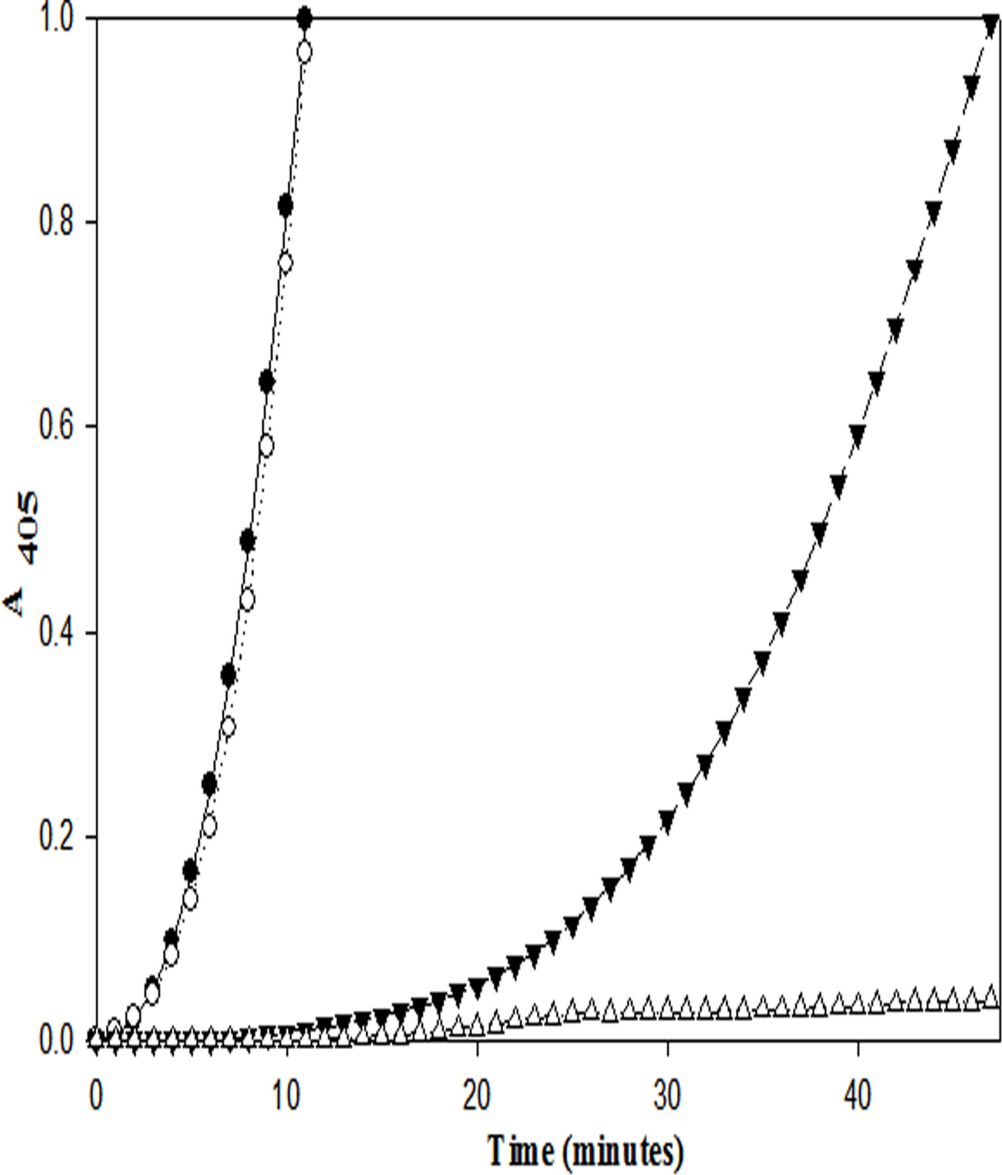
Plasminogen activation by constructs EGF-SK, SK-EGF, and SK. In these assays, 0.5 nM final concentrations of EGF-SK**(▼)**, SK-EGF**(○)** and SK**(●)**were taken in 100 μl volume in separate wells of microtiter plates (see Materials and Methods for details). Note that SK and SK-EGF show characteristic rapid plasminogen activation profiles, whereas EGF-SK exhibits a nearly 15-min lag followed by rapid plasminogen activation kinetics thereafter. Plasminogen alone in the same buffers (∆) but without any activator protein was taken as control.

**Fig 3:**
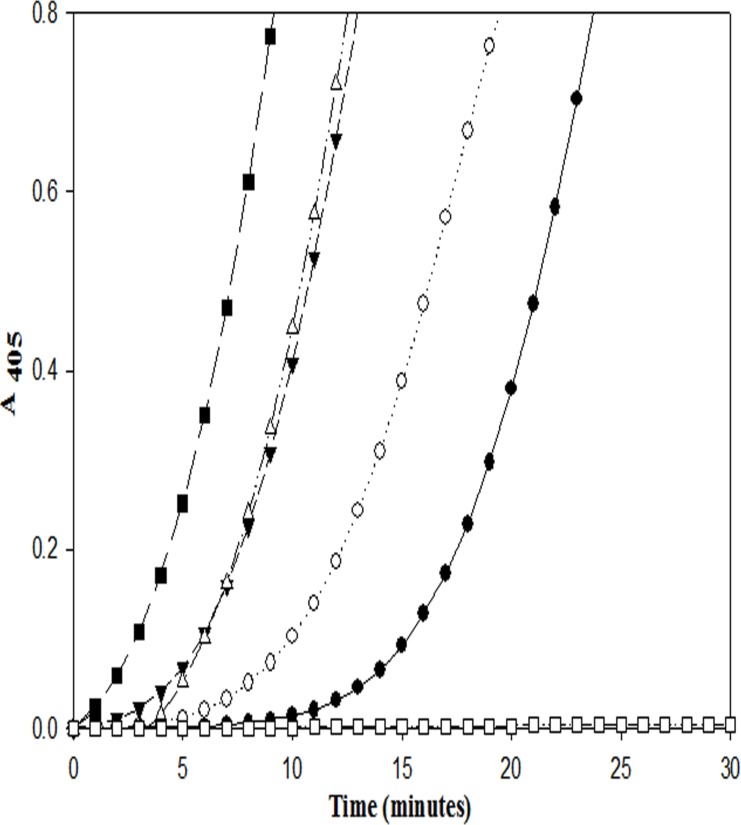
Plasminogen activation by EGF-SK in presence of increasing (trace) amounts of externally added human plasmin. In this set of experiments (see Materials and Methods for details), plasminogen activation assays using highly purified Glu-Plasminogen (2 μM) were performed with EGF-SK (0.5 nM) without plasmin (●), and with varying final concentrations of added plasmin i.e. 0.1 nM (○), 0.5 nM (▼) and 1 nM (∆). Plasminogen activation by 0.5 nM SK (■) using plasminogen (2 μM), or without any activator (□), in the absence of plasmin, were taken as controls. The respective plasmin (0.1 nM, 0.5 nM and 1 nM) controls in plasminogen without any construct/SK did not exhibit any significant change in absorbance at 405 nm (data not shown). It is notable that an increasing plasmin concentration in assays progressively decreases the lag in plasminogen activation by EGF-SK, demonstrating a plasmin-dependent activation mechanism, which is in clear distinction with native SK which does not require externally added plasmin for initiating HPG activation (see text for details regarding Pathway I and II modes of HPG activation).

**Fig 4:**
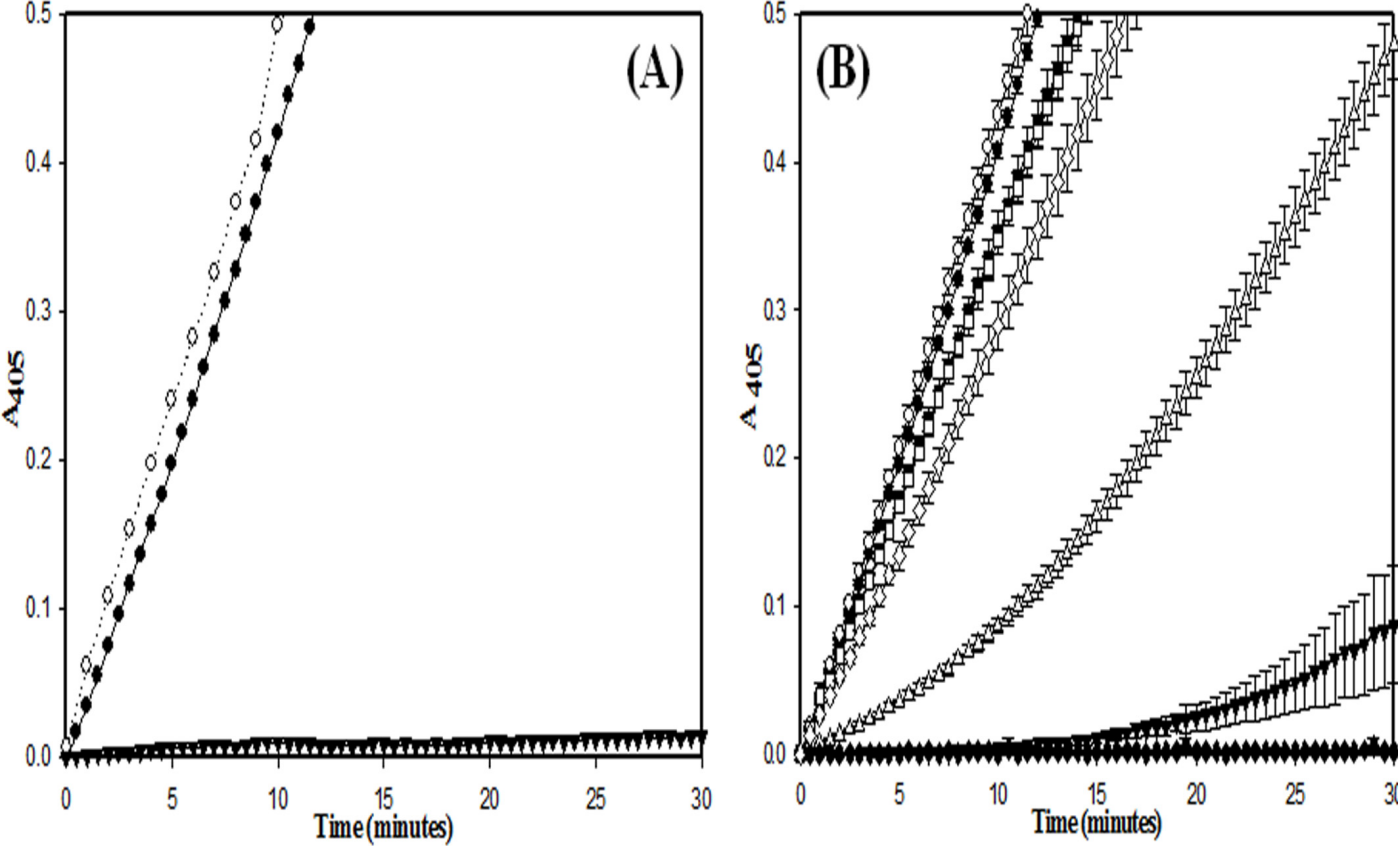
Amidolytic activation by different protein constructs. Comparison of amidolytic activation rates (kinetics of zymogen activation followed) after complexation of HPG and activator; see Materials and Methods section) formed between HPG and either SK, EGF-SK or SK-EGF fusion constructs. In panel (A), SK (●), EGF-SK (▼) and SK-EGF (○) were allowed to form complexes with Glu-HPG in separate reaction sets, and 100 nM of each complex transferred into wells of Elisa plates in final 100μl of assays containing 50 mM Tris.Cl, pH 7.4, 0.5mM chromogenic substrate (Chromozym-PL) and 0.05% BSA at 4°C. The amidolytic activation was followed by recording change in absorbance over time at 405 nm. Note that EGF-SK did not show activator complex formation (amidolytic activity) at the low temperature, whereas SK and SK-EGF showed activator species that cleaves chromogenic substrate, indicating absence of Pathway I in the former construct [5]. Similar assays were carried out at 37°C (Panel B) but 10 nm of complex was transferred to above-mentioned assay buffer composition that was incubated at 37°C (instead of 4°C), and final reaction volume was 100 μl. In Panel B, SK (○), SK-EGF (●) and EGF-SK (▼) reactions show complex formation with HPG, whereas EGF-SK:HPG complex containing 1 nM Plasmin (added along with HPG before mixing with EGF-SK) (∆), EGF-SK:HPG complex containing 10 nM Plasmin (◊), EGF-SK:HPG complex containing 50 nM plasmin (■) and EGF-SK:HPG complex containing 100 nM Plasmin (□) show accelerated amidolytic activation. This shows that at even at 37°C, EGF-SK activates only after approximately 30 min, but as plasmin (externally added) concentration increases in the reaction milieu, it shows a progressively more rapid amidolytic activation.

Both the purified proteins were subjected to human plasminogen activation assays using a single-stage continuous scanning method (see Materials and Methods), and their kinetics were compared with that of streptokinase. SK-EGF and SK exhibited closely similar plasminogen activation kinetics (Fig 2) whereas EGF-SK showed a distinct initial lag of 12–15 minutes’ duration in plasminogen activation, following which the rates became similar to that of SK and SK-EGF. In another experiment, when same plasminogen activation assays were carried out with EGF-SK, but after inclusion of trace (nanomolar) amounts of human plasmin, the lag time in plasminogen activation was found to be progressively decreased as the plasmin concentration increased (see Fig 3). When a 1:1 equimolar complex with Glu-plasminogen was made with different constructs (SK-EGF, EGF-SK or SK), and then the rates of amidolytic activation of the complexes were followed at 4°C, it was clear (see Fig 4A) that SK and SK-EGF showed characteristic amidolysis i.e. activation of ‘partner’ HPG to form an amidolytically competent active site [5], whereas EGF-SK was found incapable of doing so. This clearly indicates that the EGF-SK construct preferably follows a plasmin-mediated plasminogen activation pathway, having lost the capability to carry out the Pathway I mediated zymogen activation spontaneously after complexation with HPG [5]. Similar assays were carried out at 37°C where SK and SK-EGF showed same amidolytic potential but EGF-SK did not show any activation up to 30 minutes (see Fig 4B). However, when trace amounts of plasmin (1 nM, 10 nM, 50 nM and 100 nM) were added into HPG before mixing with EGF-SK, rapid amidolytic activation was observed. This also confirms that EGF-SK, like SK, rapidly becomes activated in presence of plasmin. The kinetic constants (K_m_ and K_cat_) for HPG activation by EGF-SK, SK-EGF and SK after making their respective 1:1 plasmin complexes (see Materials and Methods section) clearly showed that in terms of Pathway II capability, all the proteins were closely similar (Table 1).

**Table 1:** Steady state kinetic constants of SK: PN, EGF-SK: PN and SK-EGF: PN fusion complexes for Human Plasminogen activation (n = 3; data with Standard deviation).

S. No.	Thrombolytic agent	K_m_ towards PG (μM)	K_cat_ (Min^-1^)
1.	SK	0.12± 0.039^*^	12.8 ± 0.56^#^
2.	SK-EGF	0.11± 0.009^**^	12.3 ± 0. 21^##^
3.	EGF-SK	0.12± 0.013^***^	11.2± 0.35^###^

F**or** SK

* P<0.006 and

# P<0.0001.For SK-EGF

**P<0.0034,

## P<0.0001.For EGF-SK

*** P<0.0014 and

### 0P<0.0001.

**Fig 5:**
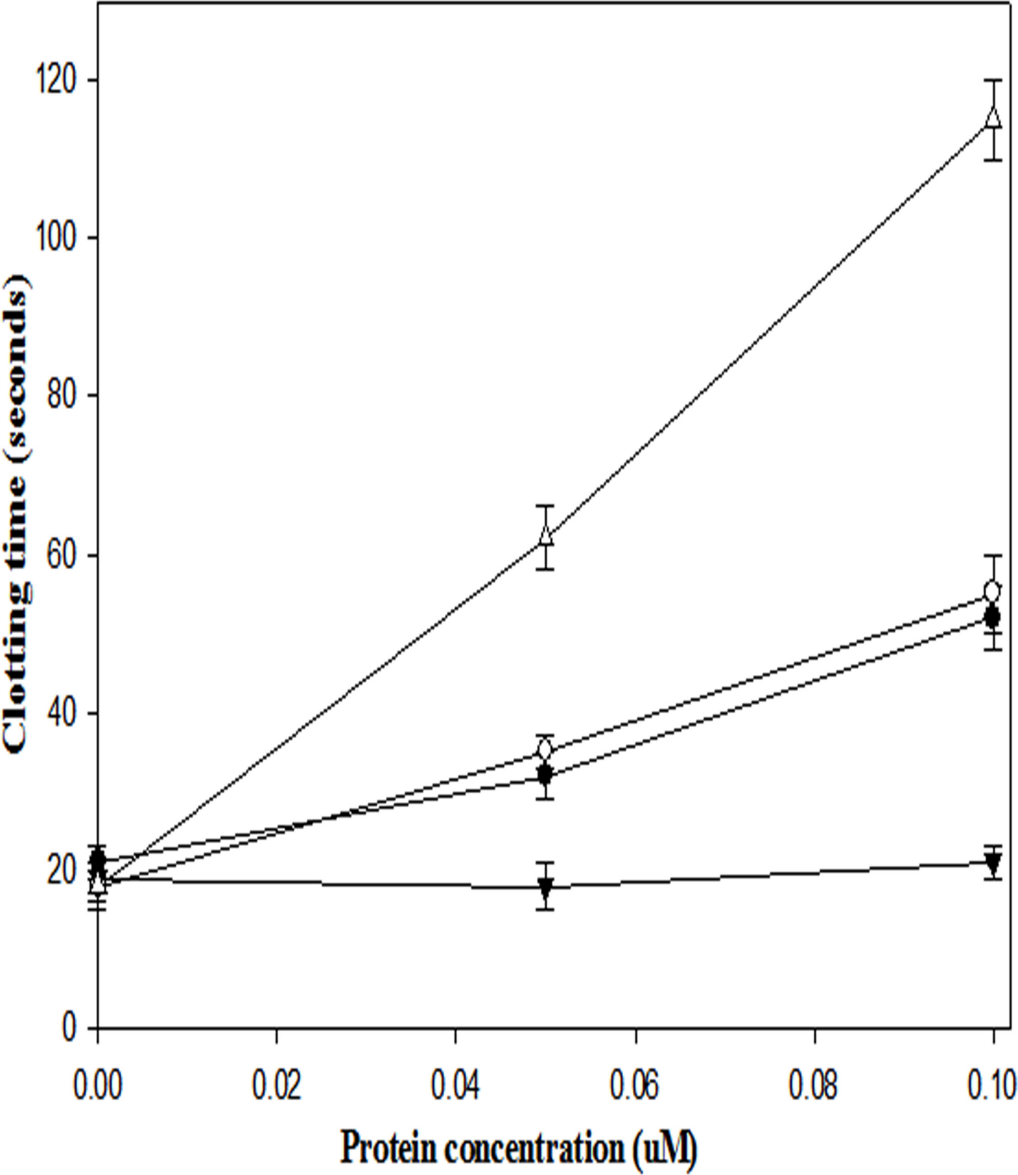
Clotting time assay in presence of different fusion constructs. In this graph (▼) represent SK; (●) represents EGF-SK; (○) represents SK-EGF and (∆) represents thrombomodulin. All the experiment performed in triplicate and their results shown with error bars. Detailed assay procedure was described in materials and methods section. EGF 4,5,6 domains of thrombomodulin known for thrombin inhibition and known for thrombin, therefore, single domain containing EGF-SK and SK-EGF were showing thrombin nearly same clotting time. For control experiment, recombinant thrombomodulin expressed in cell lines (HEK 293) was taken as control.

Anti-thrombin properties of the fusion constructs were checked in a dose-dependent manner with the help of the well-known clotting time assay [39]. In this assay 6.6 IU of thrombin gives 20 seconds’ clotting time, but after incubating with 50 nM of SK-EGF and EGF-SK fusion construct/s, in separate reactions, approximately 40 sec clotting time (double the clotting time) was observed; similarly, 100 nM concentration of both fusion constructs gave ~ 60 sec clotting time, whereas 50 and 100 nM concentrations of thrombomodulin (purchased from American Diagnostica, which was purified from HEK 293 cell line) gave ~60 seconds and ~90 seconds clotting time, respectively. SK did not show any increase in clotting time at 50 and 100 nM concentrations (see Fig 5). When EGF 4,5,6 domains were present in fusion constructs, their Km values towards thrombin were similar to that of thrombomodulin (~10 nM) but same nanomolar amounts of EGF-SK and SK-EGF exhibited ~32% and ~28% Protein C activation activity, respectively, compared to thrombomodulin (Table 2).

**Table 2:** Comparison of apparent K_m_ towards thrombin and Protein C activation capability between thrombomodulin and SK-EGF (n = 3; data with standard deviation).

S. No.	Proteins	Km for Thrombin (nM)	Km for PC (μM)	Rate of Protein C activation per nM (% of native Human thrombomodulin)
1.	Thrombomodulin	10.3 ±1.09^*^	0.29±0.08^#^	100%
2.	EGF-SK	9.2 ±2.7^**^	0.59±0.06^##^	~32.07%
3.	SK-EGF	11.1 ±3.3^***^	0.33±0.13^###^	~28.68%

For

* P = 0.0002, for

** P = 0.021 and for

*** P = 0.021. For

# P = 0.01, for

## P = 0.0004 and for

### P = 0.06.

**Fig 6:**
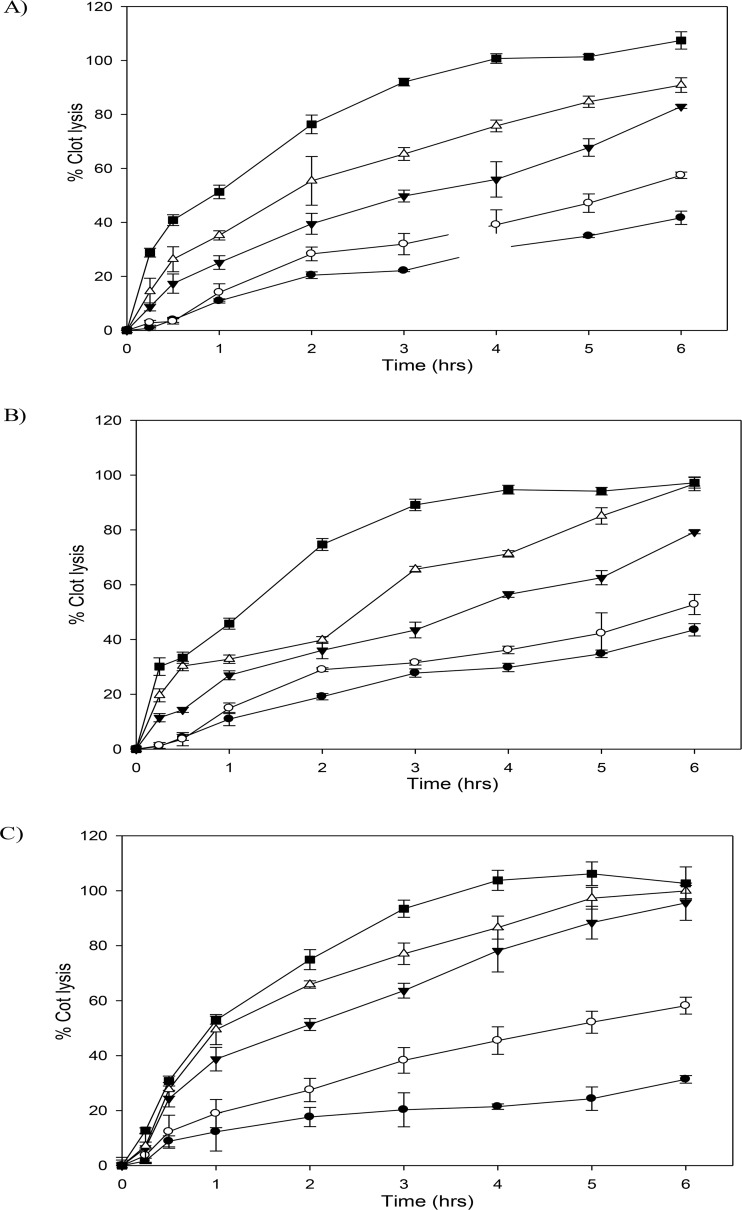
Plasma clot lysis profiles of streptokinase and their fusion constructs. In these graphs (A) represents clot lysis by SK; (B) represents clot lysis By SK-EGF (C-terminal fusion construct); (C) represents clot lysis by EGF-SK (N-terminal fusion construct). In this experiment graphs were 100 nM (■), 75 nM (∆), 50 nM (▼), 25 nM (○) and 12.5 nM (●) concentrations were used for clot lysis. Please see text for details. All the experiment performed in triplicate and their results shown with error bars.

**Fig 7:**
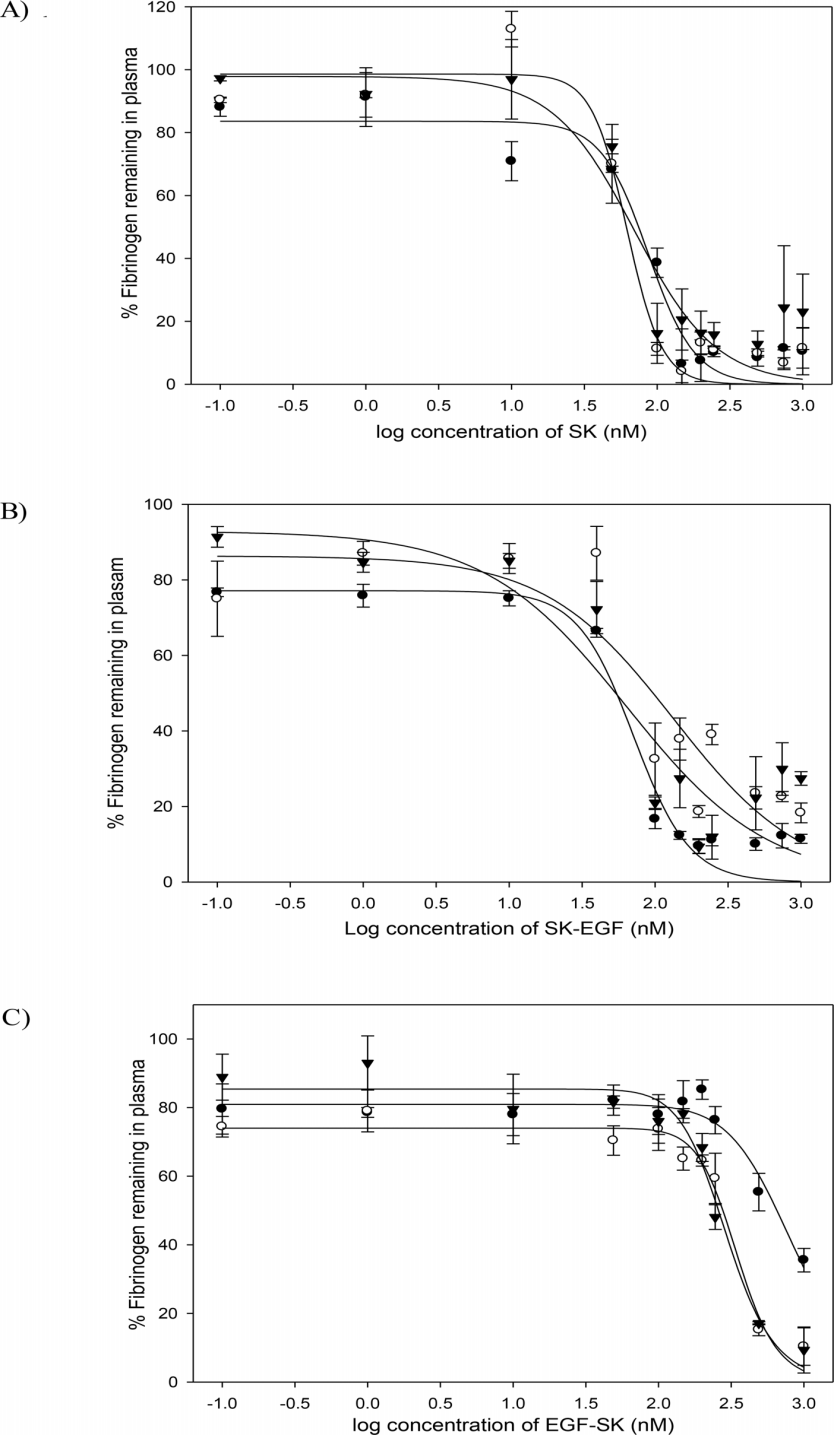
Percentage remaining soluble fibrinogen levels in plasma with different fusion activators in dose-dependent manner. Percentage of fibrinogen (Y-axis) was determined in dose dependent (X-axis; log nM concentration of different constructs) manner. In this assay, graphs A, B, and C represent remaining fibrinogen levels with SK, SK-EGF and EGF-SK, respectively; dose of different thrombolytics varied from 0.1 nM to 1000 nM. Remaining fibrinogen in plasma with each construct was determined at 15 min (●), 30 min (○) and 45 min (▼).All the experiment performed in triplicate and their results shown with error bars (P<0.0001).

Plasma clot lysis potential of different fusion constructs was then checked in human plasma by a well-established protocol [41] described in the Materials and Methods section. Briefly, this involves taking human plasma clots which contains fluorescein labeled fibrinogen and suspending these in fresh plasma to which various plasminogen activators are added. The liberation of fluorescein labeled degraded fibrin in plasma is an indicator of clot lysis that increases fluorescence of plasma as the clot lysis proceeds. Plasma clot lysis results showed that SK and C-terminal SK-EGF fusion constructs exhibited essentially the same clot lysis capabilities, whereas the N-terminal fusion, namely EGF-SK, showed a slightly rapid rate as indicated by the time corresponding to 50% clot lysis (see Fig 6). From the *in vitro* plasma clot lysis assays one can conclude that the absence of Pathway I capability ie plasminogen activation upon contact did not affect the clot lysis capability of EGF-SK, likely because the clots are enriched in plasmin [12] leading to a protection of the construct when free in plasma, but resulting effectively in a localized activation in the vicinity of the clot unlike the case of SK and SK-EGF, which did not have only Pathway II (ie plasmin dependent) mode of HPG activation. This was borne out in experiments that measured the fibrinogen protection in plasma samples where different constructs were incubated in dose dependent manner. The results (Fig 7) suggest that in case of EGF-SK which could not rapidly activate HPG, the fibrinogen in plasma was significantly protected against degradation. The estimation of fibrinogen was carried out in samples where different doses of fusion constructs had been added into plasma without the fibrin clots (see Fig 7), and their fibrinogen levels checked at various time points. In case of SK and SK-EGF, which rapidly activate plasminogen into plasmin, there is a corresponding rapid fall in the levels of soluble fibrinogen but in case of EGF-SK the fibrinogen degradation at same concentrations and times were significantly less (see Fig 7).

**Fig 8:**
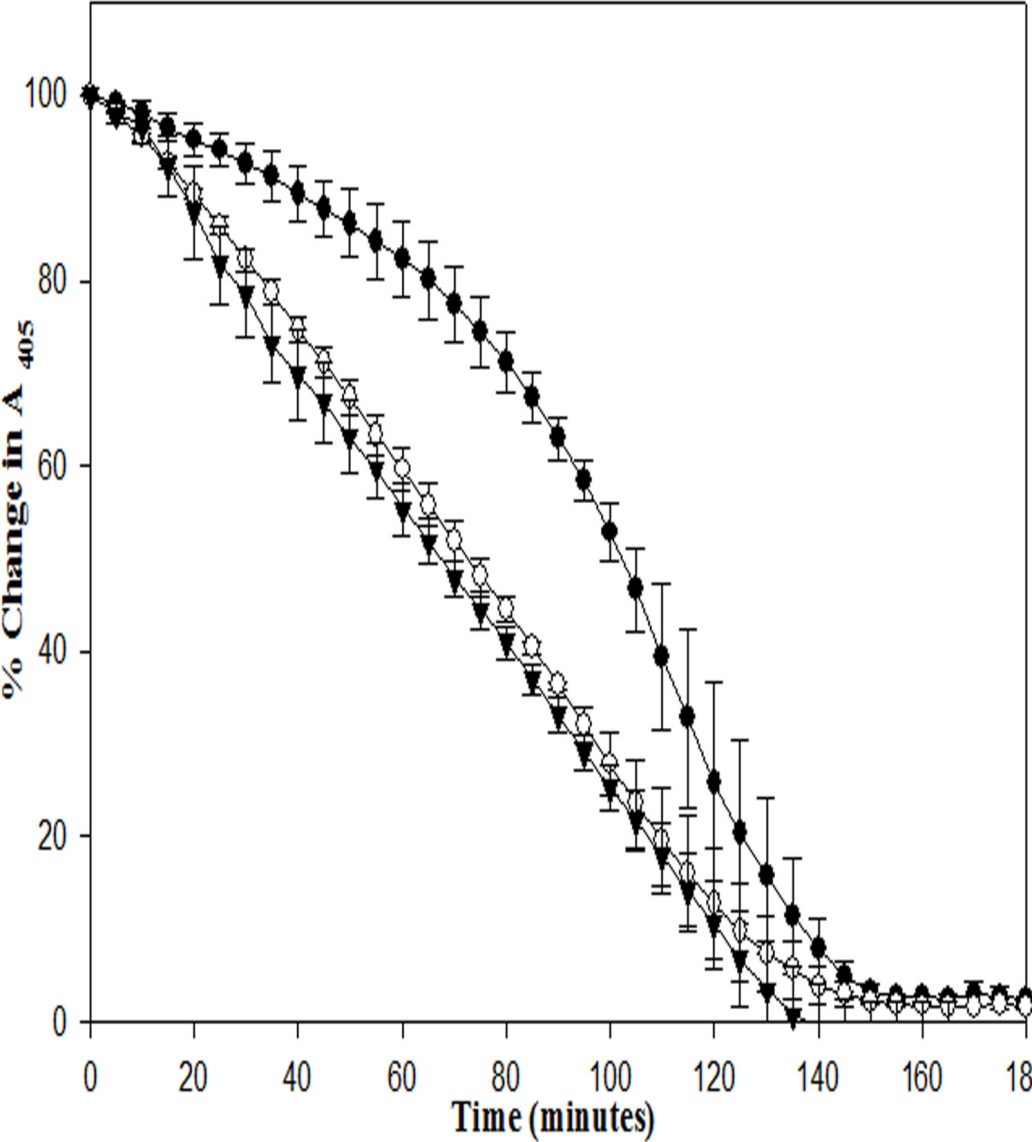
Affinity of different fusion constructs towards clot-bound thrombin. This was tested in microtiter plates by a clot lysis method that is described under Materials and Methods. In this graph, Y-axis shows relative percentage change in absorbance at 405 nm that shows fibrin degradation, and X-axis represents time in minutes. In this panel EGF-SK (○), SK-EGF (▼) show faster clot lysis compared to SK (●).All the experiment performed in triplicate and their results shown with error bars.

**Fig 9:**
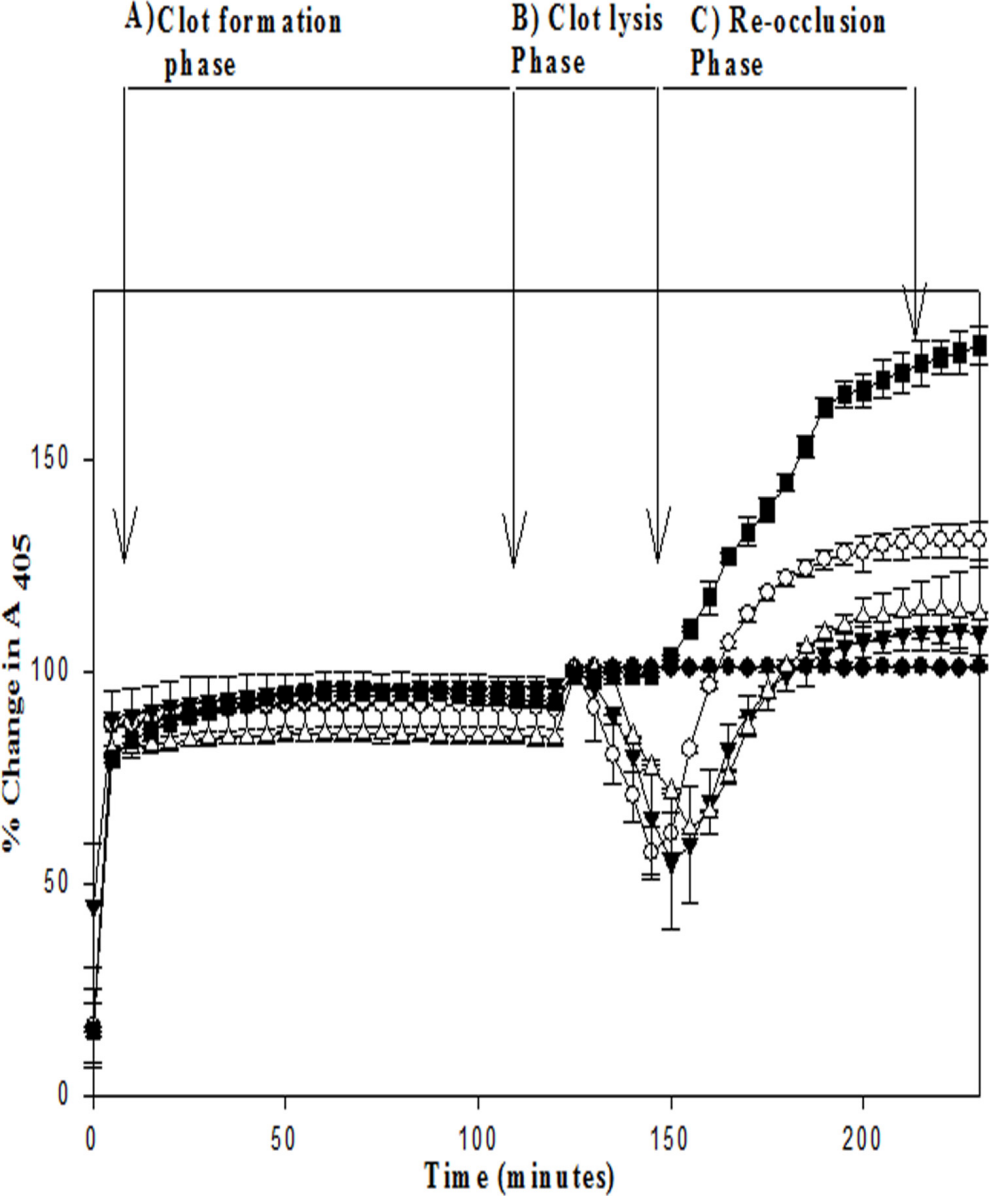
Comparative *in vitro* clot-bound-thrombin mediated re-occlusion of EGF-SK, SK-EGF and SK constructs. Re-occlusion prevention property, if any, in the various thrombolytic proteins was tested as described under Materials and Methods by a spectrophotometric assay either with SK (○), SK-EGF (∆), or EGF-SK (▼) or without any thrombolytic agent as a negative control (●). As detailed in the text, the assay was defined in three phases: a) Clot formation phase, b) clot lysis phase, and c) re-occlusion phase. It is notable that in case of EGF-SK and SK-EGF, the levels of re-clotting was ~30–40% less compared to SK (○). A HBS buffer control was also included (●) where no thrombolytic (for clot lysis phase) or fibrinogen (for re-occlusion phase) was added. In another control (■) thrombolytic was not added, but after completing time duration of clot lysis phase as seen in case of other constructs, fibrinogen and α-2 anti-plasmin were added, and change in absorbance at 405 nm was monitored. Time of washing between two phases was approximately 1–2 minutes. All the experiment performed in triplicate and their results shown with error bars.

Thus, the results clearly suggest that a plasmin-specific HPG activation mechanism (in EGF-SK) would provide significant clot-specificity likely because free plasmin in circulation is known to be rapidly inactivated by plasma Serpins, namely alpha-2-antiplasmin and alpha-2-macroglobin, whereas the fibrin clot is highly enriched in fibrin-bound, and Serpin-shielded, plasmin [12]. It is also likely that the EGF 4,5,6 domains would provide additional build-up in the fibrin clots because of their intrinsic affinity towards thrombin [45]. In order to check whether these constructs lyse clots faster due to the presence of EGF 4,5,6 domains that have a certain affinity towards thrombin, or clot-bound thrombin, clots was prepared in flat-bottomed microtiter plates as described in Materials and Methods, and comparable concentrations of constructs were incubated with preformed fibrin clots in absence of human plasminogen, after which the clots were washed with buffer (HBS), and human plasminogen was then added, and clot lysis monitored by following change in absorbance at 405 nm (see Materials and Methods). Interestingly, both EGF-SK and SK-EGF showed much rapid clot lysis compared to SK (Fig 8). This indicates that EGF 4,5,6 domains bound preferentially to clot-bound thrombin, and this resulted in significant enhancement of clot lysis rates compared to SK. In order to check whether EGF-fusion constructs could prevent reformation of clots or early re-occlusion events, a new microtiter plate based assay was developed, an *in vitro* assay system for simulating re-occlusion, which is described in the Materials and Methods section. In this assay, essentially, clots are formed in microtiter plate wells, thrombolysis initiated as before for clot lysis assays, but after ~30% clot lysis has occurred, plasminogen/plasmin was rapidly depleted from the reactions by washing. Then, trace amounts of remaining (clot-bound) plasmin was inhibited by addition of α-2-antiplamin along with addition of physiological fibrinogen concentrations to initiate clotting due to presence of clot-bound thrombin. The process of clot lysis, clot reformation etc can be easily followed through spectrophotometric measurements in real time. The obtained results (see Fig 9) indicate that EGF-SK showed 30–35% less re-conversion of fibrinogen to fibrin/ re-occlusion compared to SK. The SK-EGF (C-terminal fusion construct) also showed inhibition of reformation of clots compared to SK but less than that seen in case of EGF-SK.

## Discussion

Streptokinase and other plasminogen activators activate plasminogen to plasmin, which dissolves pathological blood clots, but hemorrhage and reformation of clot/s are major clinically challenging problems as the free HPG circulates within the system unless HPG activation is limited to the locale of the clot [46–48]. Tissue plasminogen activator shows several-fold increased fibrinolytic activity in presence of fibrin [49] which makes it the thrombolytic of choice wherever affordable, due to its higher clot specificity compared to SK and Urokinase. Tissue plasminogen activator has, over the years, been widely accepted to be the safer thrombolytic drug for myocardial infarction and stroke, but recent stroke studies and trials with tPA suggest that this agent adversely affects the integrity of the blood-brain barrier if given after a small (3–4 h) “window of opportunity.” Studies also suggest that if administration of activated Protein C is combined with tPA therapy then it reduces chances of hemorrhage during therapy in strokes [50].

The mechanism whereby SK activates the zymogen (human plasminogen) has been elegantly elucidated [5,51] revealing that residue 1 (N-terminal Ile) of SK plays a crucial role in plasminogen activation via the so-called Pathway I activation route [5]. In N-terminal deleted SK, with the initial 59 residues removed, almost no plasminogen activation capability was observed, but it showed increased plasminogen activation in presence of soluble fibrin as well as better clot lysis [52], an observation that suggests that it became a fibrin-targeted thrombolytic even without presence of any fibrin-specific domain. Similarly, anti-fibrin 59D8 antibody fused at the N-terminal of SK was also found inactive in plasma but got activated in presence of fibrin clot and showed slow clot lysis compared to SK with observable fibrinogen protection [11]. In another paper, a quite novel and interesting approach for fibrin targeting was demonstrated where N-terminal of streptokinase has been fused with a hirulog peptide (‘hirulog’ which is a part of hirudin that possesses thrombin inhibiting sequence) and a fibrin-specific antibody containing thrombin first adheres to clot and ‘attracts’ the hirulog-SK fusion towards the clot [53]. In literature, it has not been demonstrated if once the N-terminal of SK is blocked by some large bulky protein domain**/**s, then it will or will not inhibit pathway I and preferably follow pathway II, and consequently become a more plasmin-specific, clot-specific activator.

In this study, distinct plasmin-dependent activation was shown by the fusion protein EGF-SK that causes it to be targeted towards the selective dissolution of fibrin clots without a generalized degradation of fibrinogen and other blood proteins that is so often seen with streptokinase. It has been reported that plasminogen concentration is about 30-fold higher at the clot surface, and tissue type plasminogen activator and two-chain urokinase-type plasminogen activator also get attached to the surface of clots and get activated preferentially [54,55]. A relatively higher concentration of plasmin could be presumed at the locale of the clot, and a Pathway I-suppressed fusion construct is likely to activate plasminogen at or in the near vicinity of fibrin clots. This could be the probable mechanism of N-terminal deleted SK or N-terminally blocked SK which showed unexpected fibrin specificity without the presence of any fibrin-affinity domain/s. It has been clearly established that such an activator will work in a clot- specific manner, and less fibrinogen degradation will ensue under conditions where effective clot dissolution is observed. This is the case with EGF-SK, and the fibrinogen protection observed is likely to be of significant value during SK mediated therapy in the clinic. In contrast, either native SK or SK-EGF (C-terminal EGF fusion) did not show any change in activity because their N-termini are available to interact with plasminogen and initiate Pathway I of plasminogen activation. SK is normally administered as an infusion due to its tendency to activate HPG systemically, in contrast to the more clot specific (but expensive) thrombolytics such as TPA and Tenecteplase. The fact that EGF-SK is clot specific in action may allow it to be also administered as a bolus (injection) and thus position this thrombolytic as the one of choice as well as affordability.

In addition to fibrin-specificity, both thrombin inhibition and Protein C activation capabilities are seen to be surviving in SK-EGF and EGF-SK fusion constructs. However, the N-terminally fused construct (EGF-SK) was found to have a selective, plasmin-dependent mode of HPG activation, as well as significant fibrinogen protection. The fibrinogen protection observed in the *ex vivo* plasma experiments in the current study are likely to be much more quantitatively significant *in vivo* since the half -life of these constructs, like that of SK, in humans as well as test animals, is about 15–20 min. Thus, most of the free plasma proteins are majorly removed within 30–60 min after administration (unlike the *ex vivo* system in which excretion/turn-over does not happen). It is also expected that, under *in vivo* conditions during clot lysis, EGF 4,5,6 would remain bound to thrombin, and activate Protein C *in situ* and degrade the activated form of Factor V [56] and VIII [57] that promote thrombin generation. Hence, the HPG activation by the agent will likely be highly restricted to the immediate locale of the clot and likely also thwart re-occlusion due to reformation of fibrin clots. These constructs must be tested in one or two extremely selective primate species (e.g., *Cynomolgus*) in which *S*. *equisimilis* streptokinase is active [58] before being subjected to clinical studies in humans.

The obtained results on Protein C generation, clot-specific mode of action and thrombin inhibition exhibited by EGF-SK now need a detailed evaluation of this engineered molecule for ischemic stroke, apart from myocardial infarction, where, worldwide, there exists an acute and urgent need for newer and more effective thrombolyics without the side-effects seen with tPA and its new generation analogs [59].

## Supporting Information

S1 File Amino acid analysis of chimeric fusion constructs. **A).** SK-EGF Amino acid analysis. **B).** EGF-SK Amino acid analysis.
(DOCX)
