# Supplementary material for: Amino-Terminal Fusion of Epidermal Growth Factor 4,5,6 Domains of Human Thrombomodulin on Streptokinase Confers Anti-Reocclusion Characteristics along with Plasmin-Mediated Clot Specificity
Source: PLoS One. 2016 Mar 14;11(3):e0150315. doi: 10.1371/journal.pone.0150315 (PMC4790962; doi:10.1371/journal.pone.0150315)
Supplement: S1 File — A). SK-EGF Amino acid analysis. B). EGF-SK Amino acid analysis. (DOCX) [file pone.0150315.s001.docx]

**Supporting Information**

**S 1 Tables. Amino acid analysis of chimeric fusion constructs.**

A). SK-EGF Amino acid analysis.

| NAME/ID # | SK-EGF |  |  |
| --- | --- | --- | --- |
| VOL HYDROLYZED | 14µl |  |  |
| Molecular weight | 57097 |  |  |
| AMINO ACID | known Composition | Exp COMP | % ERROR |
| ASP (D) | 75 | 70 | 6.666667 |
| GLU (E) | 57 | 63 | 10.52632 |
| SER (S) | 28 | 32 | 14.28571 |
| GLY (G) | 31 | 39 | 25.80645 |
| HIS (H) | 13 | 14 | 7.692308 |
| ARG (R) | 20 | 19 | 5 |
| THR (T) | 33 | 33 | 0 |
| ALA (A) | 30 | 28 | 6.666667 |
| PRO (P) | 29 | 27 | 6.896552 |
| TYR (Y) | 20 | 12 | 40 |
| VAL (V) | 26 | 21 | 19.23077 |
| MET (M) | 5 | 2 | 60 |
| ILE (I) | 30 | 25 | 16.66667 |
| LEU (L) | 43 | 41 | 4.651163 |
| PHE (F) | 21 | 21 | 0 |
| LYS (K) | 30 | 30 | 0 |

B). EGF-SK Amino acid analysis.

| NAME/ID # | EGF-SK |  |  |
| --- | --- | --- | --- |
| VOL HYDROLYZED | 17.6µl |  |  |
| Molecular weight | 59858 |  |  |
| AMINO ACID | known Composition | EXP COMP | % ERROR |
| ASP (D) | 81 | 88 | 8.641975 |
| GLU (E) | 58 | 67 | 15.51724 |
| SER (S) | 29 | 30 | 3.448276 |
| GLY (G) | 29 | 35 | 20.68966 |
| HIS (H) | 13 | 12 | 7.692308 |
| ARG (R) | 23 | 24 | 4.347826 |
| THR (T) | 36 | 33 | 8.333333 |
| ALA (A) | 29 | 30 | 3.448276 |
| PRO (P) | 31 | 34 | 9.677419 |
| TYR (Y) | 25 | 24 | 4 |
| VAL (V) | 26 | 24 | 7.692308 |
| MET (M) | 4 | 1 | 75 |
| ILE (I) | 29 | 32 | 10.34483 |
| LEU (L) | 45 | 50 | 11.11111 |
| PHE (F) | 21 | 20 | 4.761905 |
| LYS (K) | 32 | 30 | 6.25 |

Note: Met and Tyr is sensitive to destruction during hydrolysis. Gly and Ser are often contamination comes during handling.

**Amino acid analysis of fusion constructs:**

SK-EGF (Table S. 1. A) and EGF-SK (Table S. 1. B) amino-acid analysis was carried out by Waters PICO●TAG^TM^ System. This method involves hydrolysis of protein samples in presence of hydrochloric acid, and then derivatized with phenylisothiocynate (PITC) that produces phenylthiocarbamyl (PTC) amino acid derivatives. These amino acids derivatives were analyzed by HPLC at 254 nm wavelength. Standard amino acid (provide by Pierce H standard) results were compared with samples and their moles were calculated. Cys cannot be detected with this method.
